# Fixation of Unstable Femoral Juvenile Osteochondritis Dissecans Lesions with Bioabsorbable Pins—Clinical and Radiographic Outcomes

**DOI:** 10.3390/jcm12010276

**Published:** 2022-12-29

**Authors:** Jannes Kreher, Anna-K. Tross, Felix Wuennemann, Gregor Berrsche, Christoph Rehnitz, Alexander Barié, Holger Schmitt

**Affiliations:** 1 Orthopedics and Trauma Surgery, BGU Hospital Ludwigshafen, Ludwig-Guttmann-Str. 13, 67071 Ludwigshafen, Germany; 2 Clinic for Orthopaedic Surgery, Heidelberg University Hospital, Schlierbacher Landstraße 200a, 69118 Heidelberg, Germany; 3 Institute of Diagnostic & Interventional Radiology and Neuroradiology, Helios Dr. Horst-Schmidt-Kliniken Wiesbaden, Ludwig-Erhard-Staße 100, 65199 Wiesbaden, Germany; 4 German Joint Center Heidelberg, ATOS Praxisklinik Heidelberg, ATOS Klinik Heidelberg GmbH & Co KG, Bismarckstraße 9-15, 69115 Heidelberg, Germany; 5 Clinic for Diagnostic and Interventional Radiology, Heidelberg University Hospital, Schlierbacher Landstraße 200a, 69118 Heidelberg, Germany; 6 Center for Joint Surgery and Sport Injuries, Sportopaedie Heidelberg, Clinic St. Elisabeth, Max-Reger-Straße 5-7, 69121 Heidelberg, Germany

**Keywords:** osteochondritis dissecans, osteochondral lesion, juvenile, bioabsorbable pins, SmartNails

## Abstract

Juvenile Osteochondritis Dissecans (JOCD) is a common reason for knee pain among children. The aim of this case study was to report on clinical and radiographic outcomes after fixation of an osteochondral fragment with bioabsorbable pins in children with open growth plates. We hypothesized that surgical treatment with this technique will result in good function, high rates of radiographic healing and high return to sport rates. A total of 13 knees in 12 patients (6 male, 6 female) with a median of 13 years (11, 17) were evaluated retrospectively at a minimum clinical follow-up of 24 months. Inclusion criteria were defined as open growth plates and an unstable osteochondral lesion grade III or IV. The clinical outcome was evaluated utilizing three standardized patient-reported outcome scores (Tegner Activity Scale [TAS], Knee Injury and Osteoarthritis Outcome Score [KOOS], Lysholm Score). All patients underwent magnetic resonance imaging 15 months (3, 34) after surgical treatment and defect healing was evaluated utilizing a modified version of the Magnetic Resonance Observation of Cartilage Repair Tissue (MOCART) score. Due to the small sample size, the data was reported descriptively. The interobserver variability was calculated with the Spearman rank correlation coefficient. Comparisons were made with Wilcoxon sign rank test (or sign test). At final follow-up the median KOOS Score was 98% (79.2%, 100%) and the median Lysholm Score was 94 (69, 100) points. The Tegner Activity Scale was 7 (4, 10) points preoperatively and 7 (4,10) points postoperatively (*p* = 0.5). Complete bony ingrowth occurred in 9 knees (69%), complete cartilage defect repair in 10 knees (77%) and integration to the border zone was found in 11 knees (85%) 15 (3, 34) months following surgical treatment. Fixation of osteochondral fragments with bioabsorbable pins resulted in good functional and radiographic outcomes, a high return to sport- and a low complication rate among children with open growth plates.

## 1. Introduction

Juvenile Osteochondritis Dissecans (JOCD) was recently defined by the multicenter study group “Research in Osteochondritis Dissecans of the Knee (ROCK-Group)” as an idiopathic, focal alteration of the subchondral bone prone for disruption of the adjacent articular cartilage, instability and the risk for premature osteoarthritis [1]. An unstable osteochondral fragment may result in a free body, as originally described by König in 1887 [2]. The knee is the most affected joint and osteochondral lesions are most frequently located at the medial femur condyle, followed by the ankle, elbow, hip and shoulder joints [3]. Usually, the lesion affects only one articulation, but may occur bilaterally [3].

For children between the age of 6 to 11 years the prevalence is reported to be 9/100.000 while it is 22/100.000 among young adolescents between the age of 12 to 19 years. With a ratio of 3:1 men are affected more frequently than women [4]. Overall, the prevalence among girls and young women is increasing, which is most likely due to an increased participation of women in competitive sport and thus sports-specific specialization and training load [5].

Etiology remains a subject of controversy and vascular, biomechanical-microtraumatic, hormonal, dysplastic and genetic aspects are discussed [5]. To date, many authors agree on the theory of a reduced perfusion of the bone as a result of local embolic ischemia paired with a reduced regenerative capacity with osteonecrotic zones [6].

Decision-making for surgical therapy of JOCD is based on the stability of the lesion. Surgery is recommended in stable (immobile) lesions that do not respond well to a non-operative management or unstable (mobile) lesions. Surgical therapy may be necessary for type 2 lesions (MRI classification after Dipaola et al.) onwards, especially in adolescents with almost closed growth plates [7].

Various surgical interventions such as drilling, internal fixation and salvage procedures have been described in the literature with no gold standard at this point [3].

Good to very good clinical outcomes after fixation with cannulated screws, Herbert screws, bone pegs and metal staples were demonstrated in the past [8,9]. However, metallic implants bare disadvantages such as MRI interference and the need for hardware removal and potential complications such as migration, breakage and loosening [10]. These concerns have led to the development of bioabsorbable implants.

Bioabsorbable devices are made of polyglycolic acid (PGA) or polylactic acid (PLA). PGA has a rapid degradation rate, reportedly absorbing in three months with a high incidence of foreign body reactions [11]. PLA, which was later introduced in response to the aforementioned problems with PGA devices, can take as long as six years to absorb and may place opposing cartilage at risk for damage by the implant [12,13]. Most recent devices comprise of both PGA and PLA copolymer with the aim of maximizing their beneficial effects while minimizing the inflammatory reaction elicited from degradation products.

Only few studies have investigated the short- and long-term results after bioabsorbable fixation. Kubota et al., evaluated the Lysholm score, Tegner activity scale and the KOOS score and reported about good long-term clinical results with approximately 12 years of follow-up, regardless of skeletal maturity, the size and the severity of JOCD [14].

Further studies are needed to investigate whether bioabsorbable fixation is equivalent to metallic implants.

The aim of this case study was to report on clinical and radiographic outcomes after fixation of an osteochondral fragment with bioabsorbable pins in children with open growth plates. We hypothesized that surgical treatment with this technique will result in good function, high rates of radiographic healing and high return to sport rates.

## 2. Materials and Methods

A retrospective case series was conducted with the approval of the local ethics committee (ATOS Clinic Heidelberg, Germany) and written informed consent was obtained from all patients. All patients that underwent surgical treatment with bioabsorbable pins (SmartNails, ConMed, Utica, NY, USA) between March 2009 and August 2016 were eligible for inclusion if they met the following criteria: open growth plates, unstable osteochondral lesions grade III or IV according to the classification of Guhl et al. [10] affecting one or both femoral condyles and a minimum clinical follow-up of two years. A total of 13 patients met the inclusion criteria. Of those, one patient elected against participation in the study. All surgeries were performed by the senior author with more than 15 years of professional experience.

The current classification of osteochondrosis dissecans radiologically (Berndt and Harty [15]), MR morphologically (Dipaola [16]), and arthroscopically (Guhl [10]) can be found in Table 1 [17].

### 2.1. Implant Design and Surgical Technique

#### 2.1.1. Treatment with SmartNails

The SmartNail implant is a bioabsorbable device made of Poly-L-lactic acid (PLLA) designed for the fixation of fracture fragments and OCD lesions. PLLA is a synthetic, biocompatible, biodegradable polymer. It is available in different outer diameters (1.5 and 2.4 mm) and lengths (16, 20, 25, 35, 45 mm). Following the induction of general anesthesia, the patient was placed in a supine position and was prepped and draped in a sterile fashion. A tourniquet was applied at the patient’s thigh and the affected leg was placed in a leg holder. Standard anterolateral and anteromedial portals were used for the diagnostic arthroscopy of the knee and additional portals were created if necessary. First, a thorough evaluation of the joint compartments was performed to identify the osteochondral flake as well as concomitant lesions. With an arthroscopic probe, the osteochondral defect zone was examined for defect diameter, depth and quality of the subchondral bone bed. The following surgical steps were either performed arthroscopically or in a mini-open technique. For the mini-open technique a 5 to 10 mm vertical incision was made about the affected condyle. Subcutaneous dissection was carried out, the capsule was incised and the affected condyle exposed. The bottom of the defect area was then irrigated with saline and microfractured (Pridie drilling) to obtain a bleeding subchondral surface. A cancellous bone graft was harvested from the ipsilateral iliac crest and filled into the defect zone if bony defects had a size greater than 3 to 4 mm. Osteochondral fragments were held in place with Kirschner-wires and once reduced anatomically they were fixated with one or more bioabsorbable SmartNails, depending on the size of the osteochondral lesion.

#### 2.1.2. Postoperative Treatment

Postoperatively, all patients were restricted to partial weight-bearing for four weeks and early functional mobilization with physiotherapy was recommended.

#### 2.1.3. Clinical Evaluation

Demographic characteristics and postoperative complications were assessed from the patients’ medical records. At minimum 24 months postoperatively, questionnaires were sent to the patients and clinical outcomes and return to sport rates were evaluated by three standardized and validated knee outcome scores: TAS [18], KOOS [19], Lysholm Score [18].

#### 2.1.4. Radiographic Evaluation

MRI imaging was performed at least 3 (range, 3–34 months) months after surgery with a 0.5 tesla tomograph (S-Scan Esaote Dedicated MRI, Esaote Biomedica, Genoa, Italy) and a 3.0 tesla tomograph (MRT Magnetom Verio eco 3.0 Tesla, Siemens Healthineers, Erlangen-Forchheim, Germany) in two different radiographic outpatient clinics. The MRI protocol of the knee included cartilage sensitive proton-density weighted fat-saturated (PD fs) sequences and T1 weighted sequences and regarded suitable for MOCART classification regardless of the MRI field strengths. All MRI examinations were evaluated by two musculoskeletal radiologists with 18 and 5 years of experience in musculoskeletal MRI using a modified version of the Magnetic Resonance Observation of Cartilage Repair Tissue (MOCART) score (Table 2).

The original MOCART Score was developed in 2004 by Marlovits et al. [20] to classify articular cartilage repair tissue after biological cartilage repair. The modified version takes radiographic variables that are associated with OCD into account. These variables include bone healing, defect filling and healing, integration of the border zone, environmental reaction, the surface of the reparative tissue and evidence of knee joint effusion. The MOCART score was evaluated and validated for different (lower and higher) field strengths, with initial evaluation at 1.0 T and more recently at 3.0 T. Kreitner et al. [21] reported good diagnostic performance regarding detection of knee cartilage lesions even using a low-field 0.2 T system with arthroscopy as gold standard.

#### 2.1.5. Statistics

Due to the small sample size, the median with range was used for continuous data. The interobserver variability was calculated by Spearman rank correlation coefficient and before and after comparison with Wilcoxon sign rank test (or sign test). All statistical analysis was conducted in Microsoft^®^ Excel Version 16.49 and IBM SPSS Statistics 2019, Version 26.

## 3. Results

A total of 13 patients met the inclusion criteria. Of those, one patient with both knees affected elected against participation for personal reasons. A total of 13 knees in 12 patients (92%) with a median of 13 years (11, 17) were included in this study, six (50%) men and six (50%) women. Median clinical follow-up was obtained 48 months (24, 113) and median radiographic follow-up 15 months (3, 34) postoperatively.

Surgical fixation with SmartNails was performed arthroscopically or in a mini-open technique in 12 patients, of which one patient had a bilateral procedure. Demographics are listed in Table 3.

### 3.1. Clinical Results

A total of 12 osteochondral lesions (92%) were located at the medial femoral condyle and one lesion (8%) was located at the lateral condyle. At final follow-up the median KOOS score for all patients was 98% (79.2%, 100%). The median Lysholm Score was 94 (69, 100), 77% had an excellent or good result (Lysholm score > 83). The Tegner Activity Scale was 7 (4,10) points preoperatively and 7 (4,10) points postoperatively (*p* = 0.5). The complete match was 85%. All patients returned to their preoperative sport and 96% of patients achieved preoperative level of performance. Full range of motion compared to the contralateral side was achieved in every case. Two meniscal tears at the medial meniscus in the initial MRI were treated arthroscopically along with arthroscopic fixation of the JOCD. However, the meniscus injuries were not complications of the surgical procedure. For detailed results see Table 4.

### 3.2. Complications

In one case after SmartNail fixation a partial fragmental dislocation occurred, which necessitated revision surgery with arthroscopic removal of the loose body and debridement. Subsequently, the cartilage bone fragment healed properly.

### 3.3. Radiographic Results

All images were evaluated by two musculoskeletal radiologists with 18 and 5 years of experience in musculoskeletal MRI using a modified version of the Magnetic Resonance Observation of Cartilage Repair Tissue (MOCART) score. The interobserver variability was 0.82, demonstrating a good agreement regarding to Koo et al. [22]. At final follow-up a median modified MOCART Score of 70 (55, 85) points was achieved for the total cohort. Bony ingrowth was evaluated as complete (at the same level as adjacent bone) in 9 knees (69%), defect repair and filling of the defect was rated as complete (at the same level as the adjacent cartilage) in 10 knees (77%), integration to the border zone had occurred in 11 knees (85%) and an intact repair tissue surface was present in five knees 5 (38%). In 8 knees (62%) the surface of the repair tissue was interrupted by fibrillations, fissures or ulcerations in less than 50% of the repair tissue depth. The most present tissue reactions were subchondral edema in 7 knees (54%) and effusion was found in 2 knees (15%). Detailed results are demonstrated in Table 5.

## 4. Discussion

To date, different options for the treatment of JOCD exist and the treatment procedure is stage dependent. In one of the first reports, Smillie et al., treated an osteochondral defect with a metallic pin in 1957 [23]. Cugat et al., reported about excellent short-term results after the fixation with cannulated screws in 12 patients [24]. Guhl et al., evaluated the clinical outcome after using metallic pins. Despite complications such as pin breakage, loosening and erosion through the skin good to excellent results were found for 40 out of 46 patients [10]. Arthroscopic revision surgery became necessary in 5 cases. In addition, a second surgery was required to remove the implants. In a small case series, Matsusue et al., used poly-L- lactide pins to treat three cases of OCD and 2 osteochondral fractures and demonstrated good bone union within 3 months of surgery in all cases [25].

Few studies have investigated the integration of the cartilage bone fragment after using bioabsorbable pins for unstable JOCD at the femoral condyle.

Complications associated with bioabsorbable pins are nail breakage (36.6%), effusion (38.5%), and less commonly, meniscal tears (7.7%) and chondral irregularities (13.5%) as it was demonstrated by Nguyen et al. [26].

In this study no implant breakage was found and effusions occured in 15%. Compared to Nguyen et al., a rate of 8% incomplete defect filling was found and 62% of the patients had fibrillations, fissures or ulcerations of <50% of the repair tissue depth. However, the cartilage irregularities were assessed with different techniques using the ICRS criteria and the modified MOCART score, so the results cannot be compared directly. Dines et al., reported a mean postoperative Lysholm score after 33 months of 94 (range, 78–100) after repairing an OCD with a bioabsorbable device [27] which is comparable to our findings.

The MOCART score was modified specifically for this study to evaluate the integration and healing of osteochondritis dissecans using MRI controls. To our best knowledge, no modified MOCART score version to assess the healing of JOCD has been presented yet.

Radiographic evaluation was conducted with different MR tomographs. The most recent MRI was used for the evaluation, which is the reason for the wide range of follow-up (3–34 months). Therefore, the healing processes of the cartilage bone fragment were more advanced from a MR-morphological perspective in patients with longer follow-up. Although, bony healing (bone to bone healing) should be completed after three months. However, the cartilage might take longer.

The defect size of the initial osteochondral lesions would have been of further interest, however, due to the retrospective character of this study the intraoperative descriptions were too heterogenic to be included. The authors of this article assume that it takes longer for larger defects to heal and that the modified MOCART score may thus be lower for larger defects. This would be an interesting question to clarify in further studies.

Compared to this study, there are also large varieties in post-treatment concepts such as non-weight-bearing for a minimum of 6 weeks [27] while in this case series patients were allowed to start a full load after 4 weeks. Despite a more restrictive protocol, Dines et al., demonstrated very good clinical outcomes and 100% of the patients were able to regain their preoperative range of motion and sport level 5 months after surgery.

### Limitations

Due to the usage of two different MRI devices a bias regarding accuracy is expected. The MOCART score was evaluated and validated for different (lower and higher) field strengths, with initial evaluation at 1.0 T and more recently at 3.0 T. Data on 0.5 T cartilage evaluation at the knee are sparse, however, Kreitner et al., reported good diagnostic performance (sensitivity, specificity and accuracy of 74%, 93% and 85%, respectively) regarding detection of knee cartilage lesions even using a low-field 0.2 T system with arthroscopy as gold standard [21]. However, we acknowledge that using different field strengths is suboptimal and thus a limitation of this study. Van Dyck et al., demonstrated that imaging at 3.0 T may improve detection of subtle cartilage lesions compared to lower field strengths [28], thus a future study using only 3 T imaging protocols is desirable.

The time from surgery to MRI was not standardized thus resulting in a wide range of radiographic follow-up. Due to the time dependency of healing stages, the comparison between cases is biased.

## 5. Conclusions

This study demonstrated that surgical fixation of cartilage bone fragments with bioabsorbable pins in JOCD stage III/IV at the femoral condyle is a reliable surgical procedure. Good functional and radiographic outcomes, high return to sport- and low complication rates among children with open growth plates can be achieved.

## Figures and Tables

**Table 1:** Classification of osteochondritis dissecans (OCD) [17].

X-Ray (Berndt and Harty [15])		MRI (Dipaola [16])		Arthroscopy (Guhl [10])	
Stage 1	Small area, compression subchondral bone	Type I	Thickening of articular cartilage, but no break 1a: Bone marrow edema 1b: Fluid at lesion–bone interface	Type I	Softening and irregularity of cartilage but no fragment
Stage 2	Partially detached OCD fragment	Type II	Breached articular cartilage, low signal rim behind fragment indicating attachment	Type II	Breached articular cartilage, with the fragment not displaceable
Stage 3	Fully detached OCD fragment, still in underlying crater	Type III	Breached articular cartilage, with high signal T2 changes behind fragment suggesting fluid around lesion	Type III	Definable fragment, partially attached but displaceable (flap lesion)
Stage 4	Complete detachment/loose body	Type IV	Loose body and defect of articular surface	Type IV	Loose body and defect of articular surface

The current classification of osteochondritis dissecans radiologically with X-ray (Berndt and Harty [15]), MRI (Dipaola [16]) and arthroscopically (Guhl [10]).

**Table 2:** Variables of the modified MOCART score.

Variables of the Modified MOCART Score	Maximum Score per Item
**Bony ingrowth**	
complete (on the same level as adjacent bone).	20
partial (>50% of the adjacent bone)	15
partial (<50% of the adjacent bone)	10
unstable	0
**Degree of defect repair and filling of the defect**	
complete (on the same level as adjacent cartilage)	20
hypertrophy (above the level of the adjacent cartilage)	15
incomplete (under the level of the adjacent cartilage; underfilling) > 50% of the adjacent cartilage	10
incomplete (under the level of the adjacent cartilage; underfilling) < 50% of the adjacent cartilage	5
subchondral bone exposed (complete delamination or dislocation and/or loose body)	0
**Integration to the border zone**	
complete (complete integration with adjacent cartilage)	15
incomplete integration with adjacent cartilage (demarcating border visible; split like)	10
defect visible < 50% of the length of the repair tissue	5
defect visible > 50% of the length of the repair tissue	0
**Reaction of surrounding tissue**	
subchondral edema (no/yes)	5/0
subchondral cysts (no/yes)	5/0
subchondral sclerosis (no/yes)	5/0
**Surface of the repair tissue**	
surface of the repair tissue intact	10
< 50% of the repair tissue depth damaged (fibrillations, fissures, ulcerations)	5
>50% of the repair tissue depth damaged (fibrillations, fissures, ulcerations) or total degeneration	0
**Effusion (suprapatellar recess, Baker’s cyst)**	
effusion (no/yes)	5/0
	
Maximum Score modified MOCART Score	85

The modified MOCART score with its variables and the maximum scores per item. These variables include bone healing, defect filling and healing, integration of the border zone, environmental reaction, the surface of the reparative tissue and evidence of knee joint effusion. The modification is based on the original MOCART score by Marlovits et al. [20].

**Table 3:** Demographics of all included patients.

Demographics	
patients, all	12
knees, all	13
age (range, SD)	13 year (11, 17 year)
female, *n* (%)	6 (50%)
male, *n* (%)	6 (50%)
right knee, *n* (%)	8 (62%)
left knee, *n* (%)	5 (38%)
lateral femoral condyle, *n* (%)	1 (8%)
medial femoral condyle, *n* (%)	12 (92%)
Cancellous bone graft	3 (23%)

Overview of patients included in the study concerning demographic distribution, location and treatment.

**Table 4:** Clinical outcome.

	Gender	Date of Surgery	Age at Surgery	Affected Side	Modified MOCART-Score	TAS Pre- Surgery	TAS Post-Surgery	KOOS	Lysholm Score	MRT FU	Cancellous Bone graft	Complication	Treatment	Femoral Condyle	Range of Motion Flex/Ext
N1	F	27.03.09	17	R	65	5	4	85.70%	69	13	Y (iliac crest)	-	SmartNail (3)	lateral	140/0/0
N2	F	14.06.12	13	L	75	7	6	97%	99	28	N	-	SmartNail (2)	medial	150/0/0
N3	F	16.07.12	14	R	75	5	5	79.20%	83	34	N	-	SmartNail (3)	medial	150/0/0
N4	M	27.09.12	15	L	65	9	9	100%	94	6	Y (iliac crest)	-	SmartNail (4)	medial	130/0/0
N5	M	27.05.13	11	R	85	6	6	98.80%	99	12	N	-	SmartNail (2)	medial	140/0/0
N6	M	20.12.13	17	R	60	4	4	99.40%	99	6	N	-	SmartNail (2)	medial	140/0/0
N7	F	20.08.14	11	R	55	9	9	100%	100	18	N	-	SmartNail (2)	medial	140/0/5
N7	F	20.08.14	11	L	80	9	9	100%	95	18	N	-	SmartNail (2)	medial	140/0/5
N8	F	15.10.14	13	L	65	6	6	93.50%	94	33	N	fragmental dislocation	SmartNail (2)	medial	140/0/0
N9	M	23.02.15	13	R	75	7	7	90.50%	80	3	N	-	SmartNail (2)	medial	130/0/0
N10	F	28.01.16	15	R	70	10	10	94.60%	86	18	Y (tibia)	-	SmartNail (3)	medial	140/0/5
N11	M	02.06.16	15	L	70	9	9	97.60%	90	5	N	-	SmartNail (2)	medial	150/0/0
N12	M	10.08.16	13	R	85	7	7	99.40%	100	15	N	-	SmartNail (2)	medial	130/0/0

TAS = Tegner Activity Scale, KOOS = Knee Injury and Osteoarthritis Outcome Score, ROM = Range of Motion, Flex/Ext = Flexion/Extension in degrees.

**Table 5:** Variables of the modified MOCART Score.

Variables of the Modified MOCART Score	SmartNail
**Bony ingrowth**	
complete (at the same level as adjacent bone), *n* (%)	9 (69)
partial (> 50% of the adjacent bone), *n* (%)	3 (23)
partial (< 50% of the adjacent bone), *n* (%)	1 (8)
unstable, *n* (%)	0 (0)
**Degree of defect repair and filling of the defect**	
complete (on the same level as adjacent cartilage), *n* (%)	10 (77)
hypertrophy (above the level of the adjacent cartilage), *n* (%)	2 (15)
incomplete (under the level of the adjacent cartilage; underfilling) > 50% of the adjacent cartilage, *n*(%)	0 (0)
incomplete (under the level of the adjacent cartilage; underfilling) < 50% of the adjacent cartilage, *n* (%)	1 (8)
subchondral bone exposed (complete delamination or dislocation and/or loose body), *n* (%)	0 (0)
**Integration to the border zone**	
complete (complete integration with adjacent cartilage), *n* (%)	11 (85)
incomplete integration with adjacent cartilage (demarcating border visible; split like), *n* (%)	2 (15)
defect visible < 50% of the length of the repair tissue, *n* (%)	0 (0)
defect visible > 50% of the length of the repair tissue, *n* (%)	0 (0)
**Reaction of surrounding tissue**	
subchondral edema, *n* (%)	7 (54)
subchondral cysts, *n* (%)	4 (31)
subchondral sclerosis, *n* (%)	0 (0)
**Surface of the repair tissue**	
surface of the repair tissue intact, *n* (%)	5 (38)
< 50% of the repair tissue depth damaged (fibrillations, fissures, ulcerations), *n* (%)	8 (62)
>50% of the repair tissue depth damaged (fibrillations, fissures, ulcerations) or total degeneration, *n* (%)	0 (0)
**Effusion (suprapatellar recess, Baker´s cyst)**	
effusion, *n* (%)	2 (15)

Frequencies of defect repair variables of the modified MOCART Score.

## Data Availability

Not applicable.
